# Selective Adsorption-Based Separation of Flue Gas and Natural Gas in Zirconium Metal-Organic Frameworks Nanocrystals

**DOI:** 10.3390/molecules24091822

**Published:** 2019-05-11

**Authors:** Pengli Li, Yongli Shen, Dandan Wang, Yanli Chen, Yunfeng Zhao

**Affiliations:** 1 Tianjin Key Laboratory of Advanced Functional Porous Materials, Institute for New Energy Materials & Low-Carbon Technologies, Tianjin University of Technology, Tianjin 300384, China; lpl929@163.com (P.L.); panger_shen@126.com (Y.S.); 15510906816@163.com (D.W.); 2 School of Materials Science and Engineering, Tianjin University of Technology, Tianjin 300384, China

**Keywords:** UiO-66, carbon capture, gas separation, flue gas, methane

## Abstract

Carbon capture from flue gas and natural gas offers a green path to construct a net-zero emissions economic system. Selective adsorption-based gas separation by employing metal-organic frameworks (MOFs) is regarded as a promising technology due to the advantages of simple processing, easy regeneration and high efficiency. We synthesized two Zirconium MOFs (UiO-66 and UiO-66-NH_2_) nanocrystals for selective capture and further removal of CO_2_ from flue gas and natural gas. In particular, UiO-66-NH_2_ nanocrystals have a smaller grain size, a large amount of defects, and pending –NH_2_ groups inside their pores which display effective CO_2_ selective adsorption abilities over CH_4_ and N_2_ with the theoretical separation factors of 20 and 7. This breakthrough experiment further verified the selective adsorption-based separation process of natural gas and flue gas. In one further step, we used the Monte Carlo simulation to investigate the optimized adsorption sites and energy of CO_2_, N_2_ and CH_4_ molecules in the gas mixture. The significantly large adsorption energy of CO_2_ (0.32 eV) over N_2_ (0.19 eV) and N_2_ (0.2 eV) may help us to reveal the selective adsorption mechanism.

## 1. Introduction

Carbon dioxide (CO_2_) is regarded as the primary anthropogenic culprit for global warming and climate change, which is produced by fossil fuel [1]. The atmospheric CO_2_ concentration has increased approximately 300–400 ppm over the last 50 years (1960−2016) [2], and is speculated to reach more than 500 ppm by 2050 [3]. The main emission source of CO_2_ is the combustion of fossil fuels such as coal, oil, and natural gas. Carbon capture is broadly identified as possessing the great potential to play a critical role in meeting climate change targets [4]. Effective carbon capture is regarded as one key node of the net-zero emission energy system [1]. The major demand for carbon capture comes from the treatment of CO_2_ mixture gas including power-plant flue gas, raw natural gas, coal-bed gas, and biogas in which CO_2_ is in wide concentration range and is mixed with different gases. For example, about 5%–15% of CO_2_ is majorly mixed with N_2_ in power-plant flue gas, and a wide range of CO_2_ is regarded as an impurity of methane (CH_4_) for the raw natural gas (CH_4_: >90%, CO_2_: 0.5–1%) and coal-bed (CH_4_: >50%, N_2_: ~40%, CO_2_: ~1%) [5] gas as well as biogas (CH_4_: ~50%, CO_2_: ~50%) [6]. Therefore, how to selectively capture CO_2_ in a wide range of gas components is a big challenge and is considered as one of seven major challenges in the field of separation processes within chemical engineering [7].

Various CO_2_ capture technologies, including absorption, adsorption, cryogenics, and membranes, have been developed [8,9]. Currently, the benchmark industrially demonstrated process for post-combustion CO_2_ capture technology from power plants is amines solvent-based absorption. However, high energy is required in the regeneration process and the corrosive and volatile nature of the amines also presents problems [10,11]. Physisorption of CO_2_ into microporous materials has been widely studied in recent years. The heat of adsorption of CO_2_ onto porous sorbents is normally less than 50 kJ mol^−1^ which is much smaller than chemisorption, and thus, the regeneration process has low-energy consumption and is environment-friendly [12,13]. A variety of microporous adsorbents including zeolite, activated carbons, metal-organic frameworks (MOFs) [14], and covalent-organic frameworks (COFs) [15,16] have been developed. Among these porous materials, MOFs have attracted significant attention owing to their enormous variety of interesting structural topologies and wide range of potential applications. These are constructed from metal ions as nodes and multidentate organic ligands as linkers. Adsorption and further separation of CO_2_ in MOFs have been intensely studied [12,17,18,19], and a variety of strategies of metal open-site, amino-functionalization, and pore size optimization have been successfully demonstrated. However, the major drawbacks of stabilities and robust fabrication limited the further application of MOFs. UiO-66 is a metal-organic skeleton material containing Zr developed by the University of Oslo in Norway in 2008 [20]. UiO-66 and its –NH_2_ modified derivates are considered good adsorbents for molecule and ion adsorption in gas [21] and solution [22,23] due to their excellent stability in heat and water [24]. In particular, UiO-66-NH_2_, which contains amine pendant groups on benzene dicarboxylate linkers, has showed the potential for selective adsorption of CO_2_ over N_2_ or CH_4_ when simply comparing the gas adsorption performance under conditions of equilibrium [25,26,27]. UiO-66-NH_2_ nanocrystals were also used as filler to prepare a mixed-matrix membrane for CO_2_/N_2_ [28] and CO_2_/CH_4_ [29] separation. However, the carbon capture from the mixture gas is a non-equilibrium process; the dynamic research of CO_2_, CH_4_ and N_2_ selective adsorption and separation at both the experimental and the theoretical level requires more attention, which is critical to develop sustainable carbon capture technology.

In this work, we synthesized UiO-66 and UiO-66-NH_2_ nanocrystals in a fast and easy way. UiO-66 and UiO-66-NH_2_ displayed CO_2_ selective adsorption ability over N_2_ and CH_4_. The ideal selectivity of CO_2_/N_2_ and CO_2_/CH_4_ in UiO-66-NH_2_ was calculated to be 20 and 7 under 298K, respectively. The carbon capture from flue gas and raw natural gas was performed in a UiO-66-NH_2_ packed column by breakthrough experiments. CO_2_ molecules can be effectively removed with the selective factor of seven (CO_2_/N_2_, 15/85 in volume) and two (CO_2_/CH_4_, 10/90 in volume), respectively. In addition, the separation process was further simulated by theoretical calculation to recover the binding energies of gas molecules and prefer gas adsorption abilities of UiO-66-NH_2_.

## 2. Results

### 2.1. Synthesis and Characterization

UiO-66 and UiO-66-NH_2_ were synthesized in a convenient process, in which the nanocrystals were prepared in a short time (total 2.5 h) under ambient pressure without using pressure autoclave. Typically, Zirconium tetrachloride (ZrCl_4_), hydrochloric acid (HCl, 37 wt%), terephthalic acid, and *N*,*N*-Dimethylformamide (DMF) were placed in a glass vial (100 mL) and vigorously stirred for 30 min at 80 °C. After centrifugation, washing, and drying, UiO-66 and UiO-66-NH_2_ particles were then obtained.

**Figure 1:**
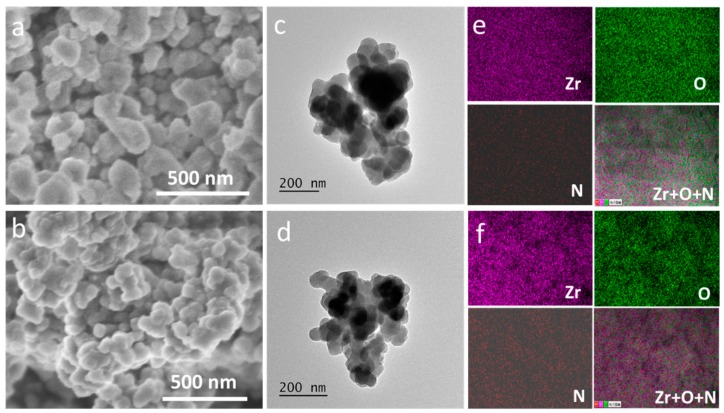
The SEM (**a**), TEM (**c**) and EDS (**e**) mapping images of UiO-66; the SEM (**b**), TEM (**d**) and EDS (**f**) mapping images of UiO-66-NH_2_.

The morphologies of UiO-66 and UiO-66-NH_2_ crystals were firstly characterized through scanning electron microscopy (SEM) and transmission electron microscopy (TEM). Figure 1a shows the morphology of UiO-66, where the typical particle size in the range of 100–200 nm was found. Synthesized UiO-66-NH_2_ possessed a smaller particle size with the typical size less than 100 nm (Figure 1b). Furthermore, the TEM images also showed that the UiO-66 (Figure 1c) and UiO-66-NH_2_ (Figure 1d) particles possess an irregular shape with the mean particle size around 200 nm (UiO-66) and approximate 100 nm (UiO-66-NH_2_), respectively, and this was mutually verified by SEM results. The energy-dispersive X-ray spectroscopy (EDS) mapping was employed to investigate the elements’ distribution. As indicated in Figure 1e,f, the elements Zr and O uniformly spread over the particles, while the element N was also founded from UiO-66-NH_2_ which is derived from the –NH_2_ group of the ligand (2-aminoterephtalic acid).

**Figure 2:**
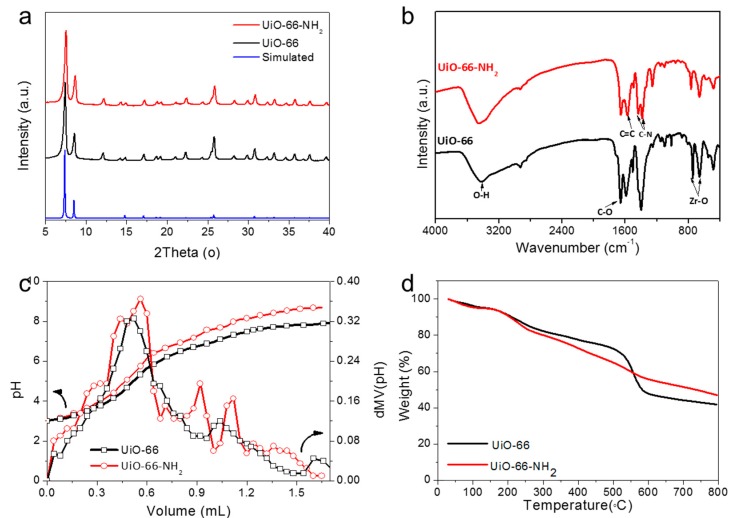
X-ray diffraction patterns (**a**), FTIR spectra (**b**), acid-base titration curves (**c**), and TGA (**d**) curves of UiO-66 and UiO-66-NH_2_.

The crystal phase was then examined by X-ray powder diffraction (XRD). Figure 2a shows the major diffraction patterns of UiO-66 and UiO-66-NH_2_, where the peaks were well consistent with the simulated pattern of UiO-66 reported previously [20]. However, the as-synthesized UiO-66 and UiO-66-NH_2_ particles exhibited broad peaks with low intensity, suggesting that some disorder and therefore large number of defects would exist in UiO-66 and UiO-66-NH_2_ [30,31]. Recent studies have shown that defects in MOFs provide a positive influence on catalysis, adsorption, and proton conductivity [32]. Fourier-transform infrared spectroscopy (FTIR) spectra in Figure 2b shows the chemical information of UiO-66 and UiO-66-NH_2._ They have similar vibrational peaks in the FTIR spectra. The characteristic peak around 3403 cm^−1^ was ascribed to the vibrational mode of the O–H group, which was related to the adsorbed water from the surface of the samples. A lot of intense peaks in the range of 1700–1200 cm^−1^ were derived from asymmetrical and symmetrical stretching vibrations of the carboxylate groups. The peaks at 800–600 cm^−1^ might be ascribed to a Zr–O bond. Especially, the peaks of 1390 and 1264 cm^−1^ were attributed to the vibrational mode of the C–N band in FTIR spectra of UiO-66-NH_2,_ which originate from the –NH_2_ group of ligands of UiO-66-NH_2_. We use acid-base titration to further determine the existence and quantity of defects in UiO-66 and UiO-66-NH_2_. The titration curves for UiO-66 and UiO-66-NH_2_ are shown in Figure 2c. There is a slow break in the curve between the pH of five and seven. To better visualize the various equivalence points, the first derivative of the titration curve is further plotted. The results show the distinct equivalence points corresponding to the pKa values in Table 1. These defects can be assigned to bridging-OH, acetic acid, and Zr–OH_2_, respectively [31]. The thermal stability was also investigated by thermal gravimetric analysis (TGA) (Figure 2d), the weight loss before 100 °C was due to the removal of adsorbed small molecules from air, ca. CO_2_ and H_2_O. No obvious decomposition was found before 500 ^o^C for UiO-66 and 300 ^o^C for UiO-66-NH_2_ indicating their superior stability [20].

### 2.2. Pore Structure and Gas Selective Adsorption

**Figure 3:**
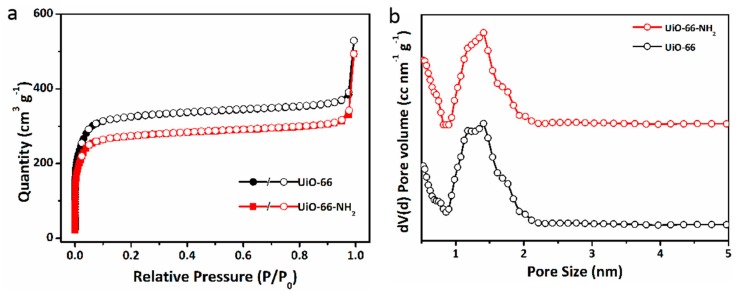
N_2_ isotherms at 77 K (**a**) and pore distribution curves (**b**) calculated from absorption curves by the NLDFT mode of UiO-66 and UiO-66-NH_2_.

The textural characteristics (surface areas, pore size and pore volume) of UiO-66 and UiO-66-NH_2_ nanocrystals are evaluated by N_2_ adsorption and desorption analysis at 77 K. The nitrogen adsorption-desorption isotherms and the pore size distribution of UiO-66 and UiO-66-NH_2_ are shown in Figure 3. The characteristic of isotherms was in accord with type-II adsorption isotherms where the primary adsorption occurred at low relative pressures <0.1 indicated the formation of a highly microporous material with the possibility of a narrow pore size distribution of UiO-66 and UiO-66-NH_2_. The adsorption curve climbed rapidly at P/P_0_ values greater than 0.95 indicating the capillary condensation derived from the aggregation of nanoparticles or defects. The results showed that UiO-66 and UiO-66-NH_2_ had a large Brunauer–Emmett–Teller (BET) surface area of 1308 and 1104 m^2^ g^−1^, respectively, and it was in good agreement with previously reported UiO-66 structures that contain defects [31,33]. The pore distributions of UiO-66 and UiO-66-NH_2_ were further investigated through the Nonlocal Density Functional Theory (NLDFT) method based on the adsorption data. The bimodal pore distributions of ultramicropores (<0.7 nm) and supermicropores (0.7–2 nm) were probed as displayed in Figure 3b. Moreover, the pore volume was 0.533 (UiO-66) and 0.462 (UiO-66-NH_2_) cm^3^ g^−1^, respectively. These results demonstrated that the prepared UiO-66 and UiO-66-NH_2_ possess a high surface area in the micropore range and thus enabled a desirable adsorption capability.

**Figure 4:**
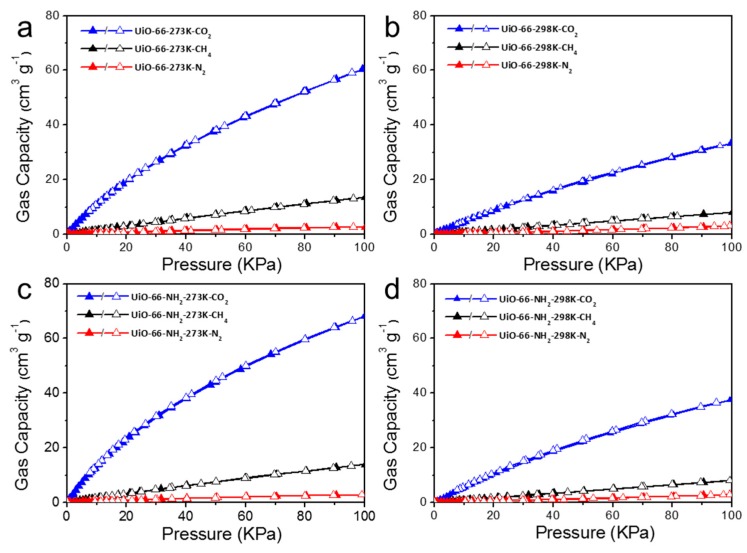
CO_2_, CH_4_, and N_2_ adsorption–desorption isotherms of UiO-66 at 273K (**a**) and 298K (**b**); UiO-66-NH_2_ at 273 K (**c**) and 298 K (**d**).

With their combination of nanosized, abundant defects and a large number of micropores, UiO-66 and UiO-66-NH_2_ demonstrated that they have great potential in the field of gas adsorption and separation. The CO_2_, CH_4_ and N_2_ adsorption-desorption curves are given in Figure 4, where the isotherms are recorded under the two temperatures of 273K and 298K, respectively. UiO-66 and UiO-66-NH_2_ exhibited excellent adsorption performance for CO_2_ at different temperatures. As shown in Figure 4, the CO_2_, CH_4_ and N_2_ equilibrium adsorption capacities of UiO-66 were 61 cm^3^ g^−1^, 13.6 cm^3^ g^−1,^ and 2.7 cm^3^ g^−1^ at 273 K and 100 kPa, respectively. For 298 K and 100 kPa, the uptake capacities of CO_2_, CH_4_, and N_2_ were 33.4 cm^3^ g^−1^, 8.1 cm^3^ g^−1^, and 3.1 cm^3^ g^−1^, respectively. The enhancement gas adsorption abilities were found from UiO-66-NH_2_. The CO_2_, CH_4_, and N_2_ equilibrium adsorption capacity of UiO-66-NH_2_ were 68 cm^3^ g^−1^, 13.9 cm^3^ g^−1^, and 2.8 cm^3^ g^−1^ at 273 K and 100 kPa, respectively. And they were 37.6, 8.1, and 2.9 cm^3^ g^−1^ at 298 K and 100 kPa. UiO-66-NH_2_ and UiO-66 have moderate CO_2_ uptakes which are comparable with MIL-100(Cr) (50) [34], UiO-66 (38) [27], MAC-4 (37.2) [35], IRMOF-1 (27.3) [36], and MOF-177 (19.8) [36] at atmosphere condition.

**Figure 5:**
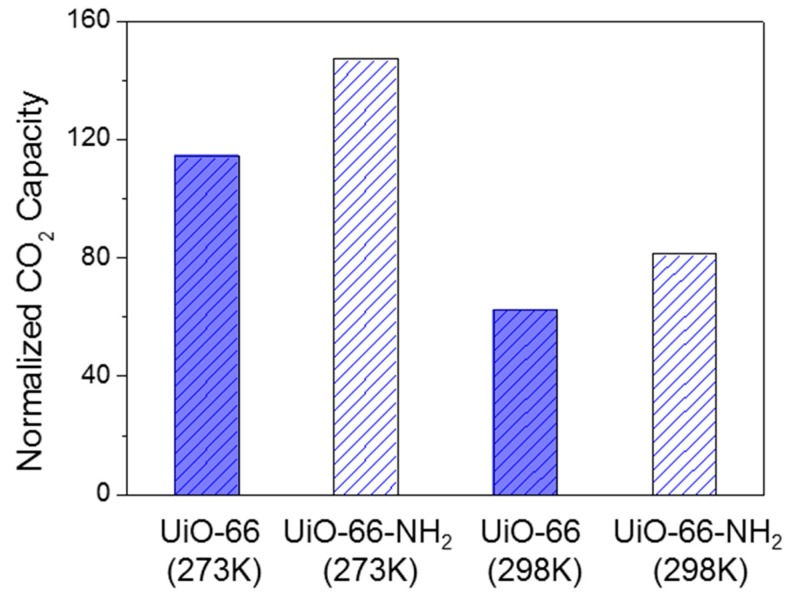
The normalized CO_2_ capacities of UiO-66 and UiO-66-NH_2_.

The CO_2_ capacity was further normalized to the pore volume to recover the affection of chemical components of UiO-66 and UiO-66-NH_2_. As indicated in Figure 5, UiO-66-NH_2_ has obvious larger normalized CO_2_ adsorption values than UiO-66. This phenomenon showed that the –NH_2_ group of ligands in UiO-66-NH_2_ may contribute more to the CO_2_ molecule adsorption sites, and this conclusion coincides with Ethiraj’s conclusion [37]. More importantly, UiO-66 and UiO-66-NH_2_ display apparent higher CO_2_ adsorption capacity than CH_4_ and N_2_ under the same temperatures and pressures, meaning that it has potential to remove CO_2_ from CH_4_ and N_2_ by selective adsorption.

### 2.3. Dynamic Separation of Flue Gas and Natural Gas

**Figure 6:**
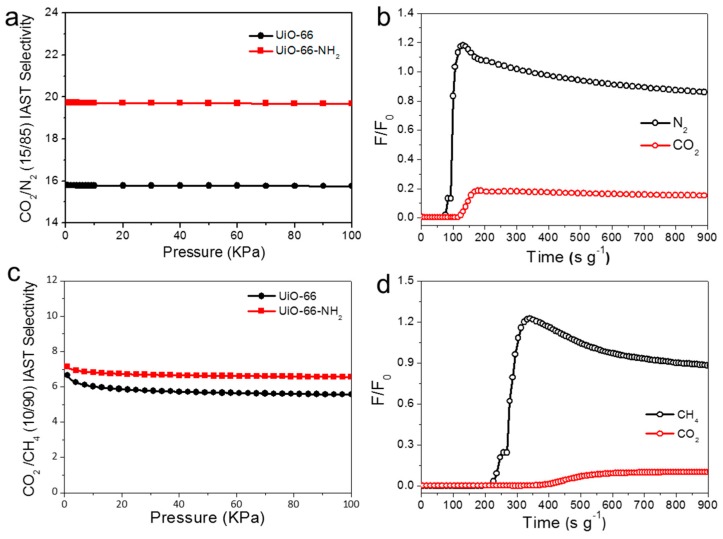
The IAST (**a**) and breakthrough separation curves (**b**) of CO_2_/CH_4_ (10/90 in volume ration) of UiO-66 and UiO-66-NH_2_; the IAST (**c**) and breakthrough separation (**d**) curves (s) of CO_2_/N_2_ (15/85 in volume ration) of UiO-66 and UiO-66-NH_2_.

The gas selective separation abilities of UiO-66 and UiO-66-NH_2_ were firstly evaluated through ideal adsorbed solution theory (IAST), which is widely used to estimate the potential of gas separation of porous materials based on single gas equilibrium adsorption curves [38]. Single-component isotherms of CO_2_/N_2_ (15/85 in volume) and CO_2_/CH_4_ (10/90 in volume) at 298 K were fitted, where the component was the typical composite of flue gas (CO_2_ and N_2_) and raw natural gas (CO_2_ and CH_4_). As shown in Figure 6, the adsorption selectivity of CO_2_/N_2_ were calculated to be about 16 (UiO-66) and 20 (UiO-66-NH_2_) at a pressure of 100 kPa and 298 K, respectively, which is comparable with UiO-66 (17.8) [25] and MOF-505 (27.8) [39]. The CO_2_/CH_4_ selectivity was about 6 (UiO-66) and 7 (UiO-66-NH_2_) at the same condition, respectively, which is at the same level with MIL-100(Cr) (8) [34], MOF-505 (7.6) [39], and MAF-66 (5.8) [40]. The IAST results indicated the feasibility of UiO-66-NH_2_ for practical application in the separation of CO_2_/N_2_ and CO_2_/CH_4_.

To evaluate the potential for real separation of the gas mixture of CO_2_/N_2_ and CO_2_/CH_4_ of UiO-66-NH_2_, the breakthrough experiments were carried out with binary mixtures of CO_2_/N_2_ (15:85, *v*/*v*) and CO_2_/CH_4_ (10:90, *v*/*v*) on a home-made column breakthrough setup (supporting information) which is the typical composition of flue gas and nature gas. As shown in Figure 6b, the results suggest the high-efficiency separation of N_2_ from 15:85 CO_2_/N_2_ by flowing the mixture gas over a packed column of UiO-66-NH_2_. It could be clearly observed that the N_2_ first breakthrough was at 7 s, while the CO_2_ could not be detected before its breakthrough point at 49 s. The separation factor was calculated to be seven following the calculation procedure provided in the supporting information. As shown in Figure 6d, the dynamic separation experiment of CO_2_/CH_4_ mixed gas (10/90 in volume ration; flow speed of 2 mL min^−1^) was also examined under room temperature (298 K). The breakthrough curves can be divided into three segments based on their adsorption characteristics. The net breakthrough times (with the dead time deducted) of CO_2_ and CH_4_ were 114 and 226 s, respectively, giving a CO_2_/CH_4_ (10/90) separation factor of about two. Therefore, the ability of selective adsorption and further remove CO_2_ from flue gas and natural gas of UiO-66-NH_2_ has been demonstrated.

### 2.4. Monte Carlo Simulation of Gas Selective Adsorption

**Figure 7:**
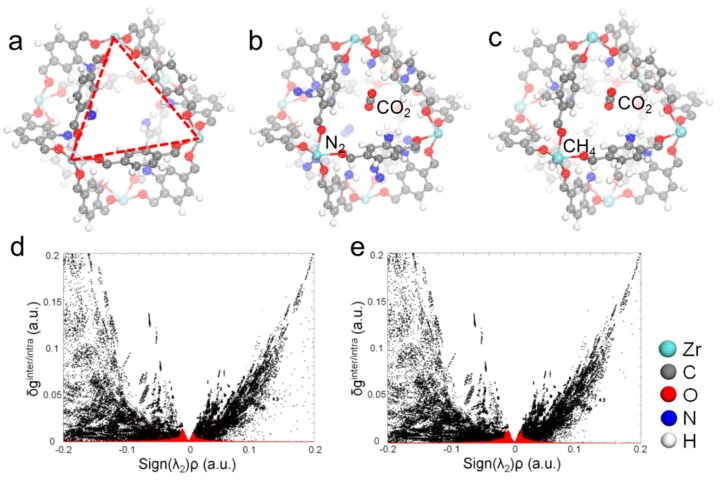
Optimized structures and intermolecular interactions between CO_2_, N_2_ and CH_4_. Stable porous cage structure of UiO-66-NH_2_ (**a**), stable adsorption structure for CO_2_ and N_2_ adsorption in UiO-66-NH_2_ (**b**), stable adsorption structure for CO_2_ and CH_4_ adsorption in UiO-66-NH_2_ (**c**), scatter plot for δ function versus sign(λ_2_)ρ of CO_2_ and N_2_ (**d**) and CO_2_ and CH_4_ (**e**) adsorption in UiO-66-NH_2_.

A simple MC simulation was further carried out to analyze the distribution position and adsorption energy of CO_2_, N_2_ and CH_4_ in UiO-66-NH_2_. The simulation results showed that CO_2_, N_2_, and CH_4_ molecules were mainly distributed in the cage surrounded by three ligands of UiO-66-NH_2_ (Figure 7a). At the initial state, one CO_2_ molecule and seven N_2_ or CH_4_ molecules were placed in the cage to follow the chemical components of flue gas and raw natural gas, respectively. The optimized structures for CO_2_ and N_2_ or CO_2_ and CH_4_ are shown in Figure 7b,c, respectively. Small molecules were found to be located in the middle of the triangle area which implies that the weak interactions may rest between small molecules and UiO-66-NH_2_. To prove these weak intermolecular interactions, an Independent Gradient Model [41] was carried out for those two structures in Figure 7b,c. The scatter plots for the δ function versus the sign(λ_2_)ρ including intermolecular (red area) and intramolecular (black area) interactions were shown in Figure 7d,e, where the sign(λ_2_)ρ is the sign of the second largest eigenvalue λ_2_ of the electron-density Hessian matrix multiplied by the electron density. It could be seen that the electron density of intermolecular interaction is not very large, but not very close to zero either. Based on this we can speculate that the intermolecular interactions in those two systems are weak interactions. The adsorption energy for CO_2_, N_2_, and CH_4_ are estimated to be 0.32, 0.19, and 0.20 eV, respectively. It can be speculated that the CO_2_ and CH_4_ or CO_2_ and N_2_ mixed gases can be effectively separated by this MOF material, which is consistent with the experimental results.

## 3. Materials and Methods

### 3.1. Chemicals and Characterizations

The chemicals used were Zirconium chloride (ZrCl_4_, ≥99.5%), terephthalic acid (99%), 2-aminoterephtalic acid (99%), hydrochloric acid (HCl, 5%), and *N*,*N*-Dimethylformamide (DMF). All chemicals were used as received without further purification. Powder X-ray diffraction patterns (XRD) were recorded on a Rigaku Ultima Iv X-ray diffractometer (Cu Kα, λ= 1.5406 Å, Rigaku, Tokyo, Japan,) at a scan rate of 10° min^−1^ in the 2θ range from 3° to 80°. Fourier transform infrared spectroscopy (FTIR) spectra were measured using Frontier MidIR FTIR (PerkinElmer, Waltham, MA, USA) with the KBr pellet technique in the range 400–4000 cm^−1^. The morphologies of the materials were observed using a Verios 460L scanning electron microscope (SEM, FEI, Hillsboro, OR, USA) and a Tecnai G2 Spirit TWIN transmission electron microscope (TEM, FEI, Hillsboro).

### 3.2. Synthesis of UiO-66 and UiO-66-NH_2_

A total of 0.625 g of ZrCl_4_ and 5 mL of 37% HCl aqueous solution were mixed and dissolved in 10 mL of DMF. After 30 min of ultrasonication, 0.615 g of terephthalic acid dissolved in 50 mL of DMF was added to the former solution of ZrCl_4_ and HCl, and the whole solution was further sonicated by using a batch sonication (Kunshan Ultrasonic Instruments Co., Ltd., KQ-100, Kunshan, Jiangsu, China) with the output power of 100 W and the frequency of 40 kHz for the next 30 min. The solution was then kept in a 100 mL glass vial at 80 °C statically without stirring or ultrasonicating at 80 °C for 2 h. UiO-66-NH_2_ was prepared following the same process except that 2-aminoterephtalic acid was used to replace the terephthalic acid.

### 3.3. Acid-Base Titrations

A total of 40 mg of sample (activated for 12 h at 150 °C) was added to a 100 mL beaker. An equivalent volume of a 0.01 M NaNO_3_ solution was added and allowed to equilibrate for 18 h. Preceding each titration, a stir bar was added to the beaker and the pH was adjusted to a value of 3.00 with 0.1 M HCl. Following this, the solution was titrated with 0.1 M NaOH of solution (adding 0.04 mL NaOH solution at a time and stirring evenly) with a pH value of 8.

### 3.4. Gas Adsorption Measurement

The N_2_ sorption isotherms at 77 K and the gas adsorption isotherms of CO_2_, CH_4,_ and N_2_ at two different temperatures (273 and 298 K) were measured by using a Autosorb-iq3 surface area and porosimeter analyzer (Quantachrome, Boynton Beach, FL, USA). The temperatures (273 and 298 K) were controlled by means of a circulating bath. The samples were degassed at 473 K for 10 h under a vacuum of 10^−5^ mmHg before the measurements. The pore size distributions and micropore surface areas were determined using the nonlocal density function theory (NLDFT) method. Gases with a high purity of over 99.995% were used.

### 3.5. Breakthrough Experiments

The breakthrough experiments of flue gas and natural gas separation were conducted in a home-made apparatus as illustrated in our previous reports [42,43].

The absolute adsorbed amount of gas *i* (*q_i_*) was calculated from the breakthrough curve by the equation:

$$q_{i}=\frac{F_{i}\times t_{0}-V_{dead}-\int_{0}^{t_{o}}F_{e}\Delta t}{m}$$

where *F_i_* = influent flow rate of the specific gas (cm^3^ min^−1^); *t*_0_ = adsorption time (min); *V_dead_* = dead volume of the system (cm^3^); *F_e_* = effluent flow rate of the specific gas (cm^3^ min^−1^); *m* = mass of the sorbent (g).

The selectivity of the breakthrough experiment is defined as α = (q_1_/y_1_)/(q_2_/y_2_), where y_i_ is the mole fraction of gas i in the gas mixture.

### 3.6. DFT Calculations

Monte Carlo (MC) simulations are carried out with the adsorption locator module with the universal force field [44]. All the geometric optimization calculations were performed using the generalized gradient approximation (GGA) with the Perdew–Burke–Ernzerhof (PBE) functional [45] as implemented in the all-electron DMol^3^ code [46,47]. The double numerical plus polarization (DNP) basis set was used throughout the calculations. The convergence criteria were set to be 2 × 10^−5^ Ha, 0.004 Ha Å^−1^, and 0.005 Å for the energy, the force, and the displacement convergences, respectively. A self-consistent field (SCF) density convergence with a threshold value of 1 × 10^−5^ Ha was specified. Independent Gradient Model analysis were carried out using Multiwfn software [48]. A complete MOF channel structure was cut-off from the single-crystal structure of UiO-66. All dangling bonds in the MOF structure (Zr atoms) were saturated by hydroxy groups.

## 4. Conclusions

In summary, we synthesized crystals of UiO-66 and UiO-66-NH_2_ in nano-size with a high surface area and abundant defects. UiO-66 and UiO-66-NH_2_ have selective gas adsorption ability of CO_2_ over CH_4_ and N_2_. The pure N_2_ and CH_4_ can be obtained from the simulated flue gas (CO_2_/N_2_, 15/85) and from raw natural gas (CO_2_/CH_4_, 10/90) by a breakthrough operation, respectively. Especially, the separation factors of seven (CO_2_/N_2_) and two (CO_2_/CH_4_) were calculated from UiO-66-NH_2_ indicating the potential applications for green separation. The results of MC simulation showed that CO_2_ displayed preferential adsorption energy over N_2_ or CH_4_ in the gas mixture through UiO-66-NH_2_. This dynamic study from theoretical and experimental aspects may provide an insight into the selective adsorption and separation of the gases.

## Figures and Tables

**Table 1:** Calculated pKa’s and corresponding equivalence volumes for UiO-66 and UiO-66-NH_2_ samples that were analyzed by acid-base titration.

MOFs	Bridging–OH		Acetate		Zr-OH_2_	
	pKa	Equi. vol. (mL)	pKa	Equi. vol. (mL)	pKa	Equi. vol. (mL)
UiO-66	3.47	0.52	5.04	1.04	6.47	1.60
UiO-66-NH_2_	3.82	0.56	4.92	0.92	5.76	1.12
